# Prevalence of covid-19 among patients with chronic obstructive pulmonary disease and tuberculosis

**DOI:** 10.1080/07853890.2022.2160491

**Published:** 2023-01-03

**Authors:** Muhammad Muneeb Hassan, Muhammad Ameeq, Farrukh Jamal, Muhammad H. Tahir, John T. Mendy

**Affiliations:** a Department of Statistics, The Islamia University, Bahawalpur, Pakistan; b Department of Mathematics, School of Arts and Science, University of The Gambia, Serekunda, The Gambia

**Keywords:** Chronic obstructive pulmonary disease, tuberculosis, SARS- CoV-2, COVID-19

## Abstract

**Background:**

The exhaustive information about non-communicable diseases associated with COVID-19 and severe acute respiratory syndrome corona virus-2 (SARS-CoV-2) are getting easier to find in the literature. However, there is a lack of knowledge regarding tuberculosis (TB) and chronic obstructed pulmonary disease (COPD), with numerous infections in COVID-19 patients.

**Objectives:**

Priority is placed on determining the patient’s prognosis based on the presence or absence of TB and COPD. Additionally, a comparison is made between the risk of death and the likelihood of recovery in terms of time in COVID-19 patients who have either COPD or TB.

**Methodology:**

At the DHQ Hospital in Muzaffargarh, Punjab, Pakistan, 498 COVID-19 patients with TB and COPD were studied retrospectively. The duration of study started in February 2022 and concluded in August 2022. The Kaplan–Meier curves described time-to-death and time-to-recovery stratified by TB and COPD status. The Wilcoxon test compared the survival rates of people with TB and COPD in two matched paired groups and their status differences with their standard of living.

**Results:**

The risk of death in COVID-19 patients with TB was 1.476 times higher than in those without (95% CI: 0.949–2.295). The recovery risk in COVID-19 patients with TB was 0.677 times lower than in those without (95% CI: 0.436–1.054). Similarly, patients with TB had a significantly shorter time to death (*p*=.001) and longer time to recovery (*p*=.001).

**Conclusions:**

According to the findings, the most significant contributor to an increased risk of morbidity and mortality in TB and COPD patients was the COVID-19.

**KEY MESSAGES**
- SARS-Cov-19 is a new challenge for the universe in terms of prevention and treatment for people with tuberculosis and chronic obstructive pulmonary disease, among other diseases.
- Propensity score matching to control for potential biases.
- Compared to hospitalized patients with and without (TB and COPD) had an equivalently higher mortality rate.

## Introduction

The worldwide COVID-19 pandemic burden had reached approximately 63 billion cases as of November 2022, with massive breakouts in different countries [1]. Chronic obstructed pulmonary disease (COPD) affected over 251 million people worldwide, and 3.17 million deaths were reported each year [2,3]. According to the World Health Organization (WHO) report 2022, based on electronic data collected from 215 countries, approximately 40 million people were treated for multidrug and rifampicin-resistant tuberculosis (MDR/RR-TB) worldwide, with 649,000 adults and 17,700 children receiving treatment [4].

Global TB prevention and treatment were challenged by the current severe acute respiratory syndrome corona virus-2 (SARS-CoV-2) pandemic [5]. According to their study, patients with COVID-19 and TB had a higher risk of dying and a lower chance of recovering. Potential treatments must target TB patients to reduce the overall impact of COVID-19. Both the treatment and prevention programs for TB had been disrupted as a consequence of the epidemic [6]. Estimates from conservative models indicated that the pandemic would likely increase TB-related deaths by 20% over the next five years [6,7] as well as the underlying causes of simultaneous COVID-19 and TB disease mortality, which not yet well understood clinically and immune pathologically [8–11]. In 2020, the first pilot study of the Global TB Network (GTN) published 49 co-infected TB/COVID-19 patients from eight countries [12], which indicated that TB diagnosed concomitantly with or after COVID-19 may enhance case-fatality.

Pakistan, a country with a lower-middle income and a population of approximately 200 million, had a high prevalence of chronic respiratory diseases, such as COPD. In Pakistan, it was estimated that the mortality rate among males due to chronic respiratory diseases was 138.2 per 100,000, while the mortality rate among females due to these diseases was 41.3 per 100,000. A global survey (BREATHE) found that 2.1% of Pakistani adults over 40 years were diagnosed with COPD [13,14]. According to the WHO report for 2022, Pakistan developed 2.8% TB of the total population, an increase of 7.9% over the previous year reported [4]. So far, there has been no study of a large multivariate link between TB and COPD in COVID-19 patients.

## Materials and methods

### Sample size and study population

A retrospective study was done at the DHQ Hospital Muzaffargarh (covering rural and urban areas of four tehsils: Muzaffargarh, Jatoi, Ali Pur and Kot Addu) to test 498 male and female patients for COPD and TB, while the total population of District Muzaffargarh was 43,48,549 [15]. According to the statistical officer at the DHQ Hospital Muzaffargarh, patient history remained in 2020–2021; the total number of TB and COPD (OPD patients), TB and COPD (indoor and follow-up cases), TB and COPD (suspected patients) and TB/COPD (conformed patients with COVID-19) were 22,240, 7514, 17,896 and 1080, respectively. Using a simple random technique, we included COVID-19 cases reported from January 2020 to December 2021. The laboratories accredited by the Punjab Health Council (PHC) and the Research Institute of Nishtar Medical College and University Multan performed real-time reverse transcription polymerase chain reaction (RT-PCR) to confirm the COVID-19 cases. COPD was diagnosed using a simple breathing test called spirometry, and in the context of pulmonary TB, sputum culture revealed *Mycobacterium tuberculosis* in the majority of reported TB patients [16]. Following a routine medical examination, the data and information were collected by a nursing staff or a consultant pulmonologist. The interview form was manually completed and collected from the resource file of the patients. This study started in February 2022 and concluded in August 2022; data collection concluded in March 2022.

### Ethics statement

The department issued a letter of ethical approval on 10 May 2022, with reference no. 1178-81/DHQ, by Aqeel Ahmad, Statistical Officer-in-charge of the ethical committee in DHQ Hospital Muzaffargarh, Punjab, Pakistan.

### Variables

This study examined various chest infection-related complaints from patients with COPD and TB. In lung diseases, COPD is a group of diseases that make it hard to breathe and block airflow, including emphysema and chronic bronchitis. The bacterium *Mycobacterium tuberculosis* is accountable for causing TB. The lungs are the most common target, but the kidney, spine and brain are all possible locations for TB bacterial infection. However, not all people who contract the tuberculosis (TB) bacteria ever develop symptoms. The SARS-CoV-2, which was discovered in 2019, is the cause of COVID-19, a respiratory disease. Coughing, sneezing and talking all contribute to the spread of the virus from person to person [17]. Diagnoses based on TB and COPD included cough, fever, sputum, right-sided chest pain, acid-fast bacillus (AFB), lower respiratory tract infection (LRTI), TB/pulmonary tuberculosis (PTB), shortening of breath (SOB), normal condition, headache, wheezing and body aches [18–29] were present.

### Outcomes

The two significant outcomes of interest elaborate on time-to-event variables.

- Death/expire
- Recovery

Both outcomes were primary, and they occurred over time. If the admitted COVID-19 indoor patients, presence or absence of TB, had severe symptoms, the case would be declared dead. If there were some clinical improvement, the patient would survive and have a better chance of recovering.

### Statistical analysis

Descriptive statistics and a Student *t*-test were used to compare the ages of patients with and without TB and COPD [30]. First, analysis was performed to estimate the relative risk (risk ratio) of mortality and recovery, and the Pearson chi-squared test was used to assess health status, gender, co-morbidities and admission status [31]. The Wilcoxon rank-sum test manipulates the survival rates of patients with TB and COPD into two groups [32]. Kaplan–Meier’s curves were used to plot time-to-event variables based on whether or not a person had TB or COPD. Patients who passed away on their last day of inpatient care were not included in the time-to-recovery analysis [33]. All statistical analyseis were done in SPSS-22 (SPSS Inc., Chicago, IL) and R (R Foundation for Statistical Computing, Vienna, Austria).

### Hypothesis

We hypothesized that COVID-19 patients with a prior history of TB or active TB would have worse clinical outcomes than those without TB. Patients with and without TB co-infection at DHQ Hospital Muzaffargarh, Punjab, Pakistan, were analysed to determine differences in mortality and recovery rates associated with the COVID-19.

Ha_1_: the median difference between TB and those who do not equal zero.

Ha_2_: the median difference between people with and without COPD is zero.

## Results

### Propensity score matching

The initially un-matched sample included 17,896 COVID-19 patients, of whom 218 (1.2%) had confirmed TB and 280 (1.56%) did not. Age significantly affected TB patients’ recovery (*p*=.001) and death (*p*=.001). The total sample’s average age was 23.81 years, with a variance of 686.50. The admitted sample created a population with similar baseline characteristics (all *p* > .05; Table 1).

**Table 1.** Demographics and health status of the matched with/without samples and matched admitted TB/COPD samples by COVID-19 status.

Matched sample	Overall (*n* = 498)	With-TB/without-COPD (*n* = 218)	Without-TB/with-COPD (*n* = 280)	*p* Value
Age (mean (variance))	23.81 (686.50)	0.44 (0.247)	0.56 (0.247)	.000
Gender (%)				
Male	385 (77.3)	170 (77.98)	218 (77.85)	.403
Female	113 (22.7)	48 (22.01)	65 (23.21)	
Survival status (%)				
Recover	399 (80.1)	167 (76.60)	232 (82.85)	.001
Death	99 (19.9)	51 (23.39)	48 (17.14)	.001
Variables (%)				
Fever	262 (52.6)	85 (38.99)	177 (63.21)	.000
Cough	355 (71.3)	108 (49.54)	247 (88.21)	.000
Sputum	232 (46.6)	102 (46.78)	130 (46.42)	.000
LRTI	45 (9)	19 (8.71)	26 (9.28)	.033
AFB	130 (26.1)	18 (8.25)	112 (40)	.001
Miller tuberculosis (MTB)/TB	134 (26.9)	26 (11.92)	108 (38.57)	.297
Normal condition	130 (26.1)	0 (0)	130 (46.42)	.007
SOB	294 (59)	30 (13.76)	264 (94.28)	.005
Body-aches	204 (41)	7 (3.21)	204 (72.85)	.406
Headache	270 (54.2)	3 (1.37)	270 (96.42)	.019
Wheezing	58 (11.6)	5 (2.29)	58 (20.71)	.507
Right side chest pain	190 (38.2)	11 (5.04)	179 (63.92)	.162

Patients with TB had a 1.476 times higher mortality rate than those without it (95% CI: 0.949–2.295). When hospitalized patients were assessed, those with TB and those without COPD had a similarly higher mortality rate. Patients had a lower chance of recovery at 0.677 (95% CI: 0.436–1.054). The estimates of RR with TB/without COPD were at 0.812 (95% CI: 0.650–1.016), and the estimates of RR without TB/with COPD observed at 1.199 (95% CI: 0.650–1.016), which denoted greater than the estimates of RR in without TB/with COPD patients (Table 2).

**Table 2.** Relative risk estimates of death and recovery with/without TB and COPD matched admitted patient samples of COVID-19.

Matched cohort	RR (95% CI)	*p* Value
With-TB/without-COPD (*n* = 218)		
without-TB/with-COPD (*n* = 280)		
Odd ratio for survival (death)	1.476 (0.949–2.295)	.083
Odd ratio for survival (recovery)	0.677 (0.436–1.054)	
For cohort with-TB/without-COPD	0.812 (0.650–1.016)	
For cohort without-TB/with-COPD	1.199 (0.963–1.494)	

**Figure 1.**
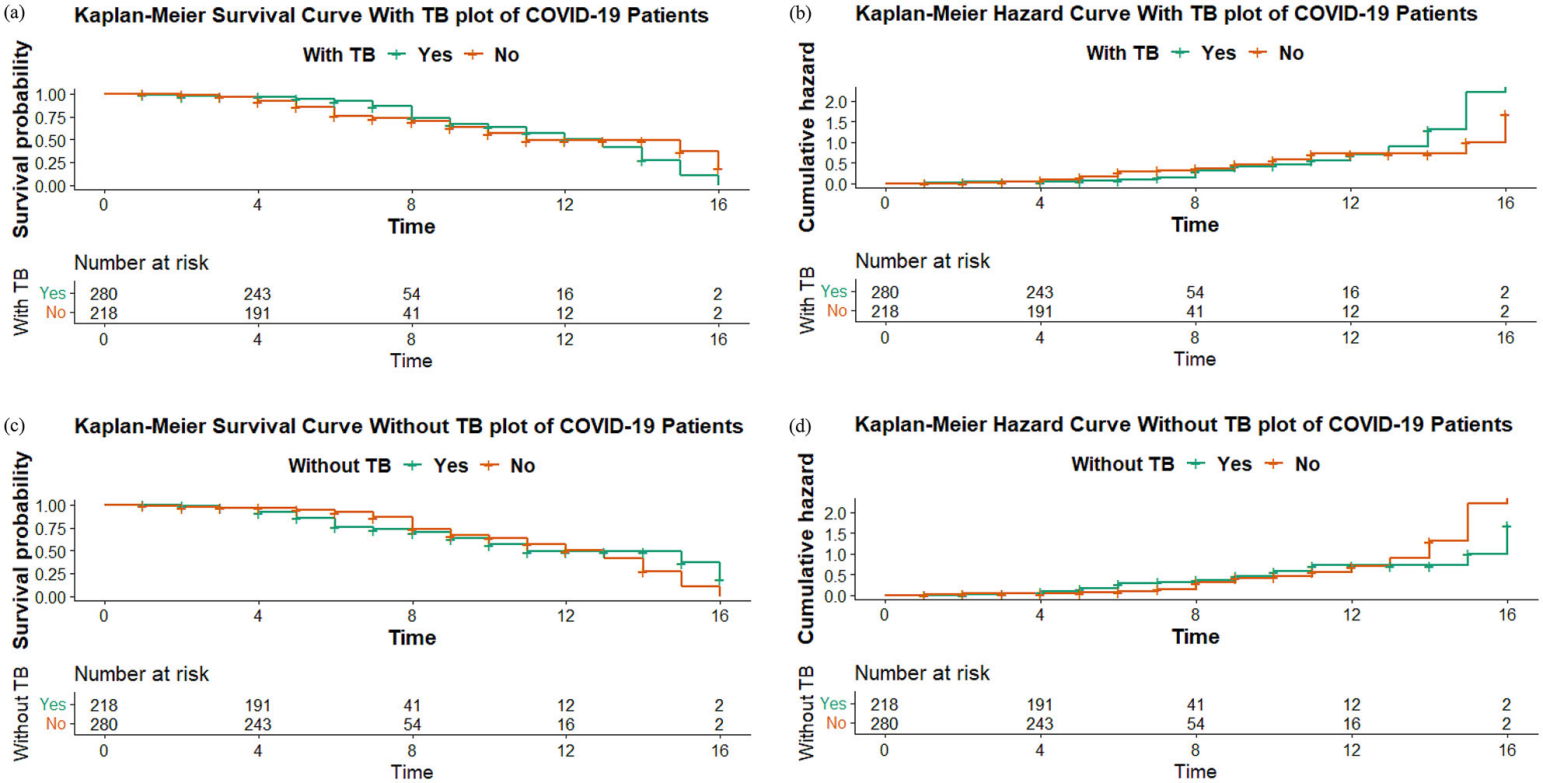
Survival analysis (a) in fully matched samples with TB, time-to-death and survival evaluated the survival function. (b) Time-to-death and survival evaluated the hazard function in fully matched samples with TB. (c) Time-to-death and survival evaluated the survival function in fully matched samples without TB. (d) Time-to-death and survival evaluated the hazard function in fully matched samples without TB.

In Figure 1, Kaplan–Meier’s curves portrayed the results pattern of the graph proposed with and without TB. In sections (a), (b), (c) and (d), the survival and hazards graphs classify the average number of patients who survive or die over time. It validates the findings that average COVID-19 patients, with or without TB, recovered in 12–16 days, and death occurred on average in 0–6 days.

## Discussion

The world has seen different diseases with time, but SARS-Cov-2 has become a new challenge for the universe in terms of the prevention and treatment of TB and COPD patients. Comprehensive measures were adopted to reduce the effect of infectious diseases accordingly. The results indicated that COVID-19 patients with TB and COPD were twice as likely to die and less likely to improve over time. On the other hand, patients with TB had shorter survival times and longer recovery times compared to patients without COPD. According to the WHO report of 3 October 2022 [34], there had been 616,951,418 confirmed cases of COVID-19 and 6,530,281 deaths were reported. Some of these fatalities can be related to TB patients with COVID-19 having higher mortality and a slower recovery rate.

Additionally, our research showed that areas with higher TB prevalence had higher COVID-19 case fatality rates. Health planning and the allocation of resources were compulsory to overcome the disease pattern. Similarly, COVID-19 cases are instantly increasing in many underdeveloped countries, which show a higher trend of TB rates and a significant difference among diseases. Fundamental strategies for individuals with TB and COPD can reduce the burden of COVID-19. Our results correspond with the findings of a study conducted in South Africa’s Western Cape Province, which revealed that COVID-19 patients with an existing TB diagnosis had a 1.47-times greater risk of death, and those with a history of TB had a 50% higher chance of death [35]. Several studies have examined the connection between COVID-19 positivity and COPD and TB. With SARS-CoV-2, TB patients are more likely to deteriorate [36].

We performed propensity score matching to account for confounding variables. However, due to residual confounding, HIV-positive COPD patients will also face difficulties. Further, there were discrepancies in the propensity scores due to the presence of non-matched co-morbid variables.

We did not have any information on HIV-AIDS in our study, which could have influenced our estimates because patients with TB and COPD and no other conditions would have made it easier to rule out other factors. In the survival analyses, we did not include people whose dates were missing. Statistical analyses were used to look at how the confounding factors spread out in the sample used for each time-to-event analysis. It ensured that there were no significant differences between patients with TB and COPD and those who did not have those diseases. Also, there might be some missing data for the primary outcomes of death and recovery. The survival analysis made up for this by censoring patients who were not the outcome on the last day of follow-up (left against medical advice (LAMA) cases).

Our findings show a direct link between COVID-19 and TB/COPD, but SARS-CoV-2 also indirectly contributes to TB and COPD morbidity and mortality. COVID-19 impacted health systems worldwide, and mortality rates for people with TB and COPD are expected to rise due to drug shortages and delays in diagnosis and treatment [37–39]. Furthermore, the inaccessibility of the health care system reduces the ability to pay for medical costs, which disproportionately affects socially disadvantaged populations. Even though the COVID-19 pandemic has caused changes to health and social systems [40], our findings show how important it is to keep TB and COPD routines and testing services a top priority. Longer follow-up studies with a large enough sample size to make inferential comparisons. Based on what we found, this study has enough participants to look into the link between COPD and TB in a high-burden COVID-19.

## Conclusions

This study found that cough, fever, sputum, right-sided chest pain, AFB, LRTI, TB/PTB, SOB, normal condition, headache, wheezing and body aches are the most common causes in COVID-19 patients with or without TB. Controlling these factors eliminates or reduces the risk of acquiring COVID-19 and SARS-CoV-2. Patients with TB had a 1.47-times greater risk of death and a 0.67 times lower chance of recovery. The study concluded that the most infective treatment combinations should be considered when designing infection prevention strategies. Our findings can help improve how COVID-19 patients’ health care is organized and help direct health interventions and protection plans to the most vulnerable chronic patients.

## Data Availability

Data, models and code supporting this study’s findings are available from the corresponding author.
